# Making It Work: The Experiences of Delivering a Community Mental Health Service during the COVID-19 Pandemic

**DOI:** 10.3390/ijerph191912056

**Published:** 2022-09-23

**Authors:** Leanne Burton, Abbie Wall, Elizabeth Perkins

**Affiliations:** Department of Primary Care and Mental Health, Institute of Population Health Sciences, University of Liverpool, Liverpool L69 3GF, UK

**Keywords:** service delivery, healthcare management, crisis leadership, crisis communication, COVID-19

## Abstract

The COVID-19 pandemic forced rapid innovative change to healthcare delivery. Understanding the unique challenges faced by staff may contribute to different approaches when managing future pandemics. Qualitative interviews were conducted with 21 staff from a Community Mental Health Team in the North West of England, UK, three months after the first wave of the pandemic. Thematic analysis was used to examine data reporting the challenges arising when working to deliver a service during the pandemic. Data is discussed under four headings; “senior trust managers trying to make it work”, “individuals making it work”, “making it work as a team”, and “making it work through working at home”. Clear communication was essential to ensure adherence to guidelines while providing safe care delivery. The initial response to the pandemic involved the imposition of boundaries on staff by senior leadership to ensure that vulnerable service users received a service while maintaining staff safety. The data raises questions about how boundaries were determined, the communication methods employed, and whether the same outcome could have been achieved through involving staff more in decision-making processes. Findings could be used to design interventions to support mental health staff working to deliver community services during future crises.

## 1. Introduction

Coronavirus 2019 (COVID-19) has impacted the population globally and was deemed a pandemic on 11 March 2020 by the World Health Organisation (WHO). Countries were advised to implement measures such as social distancing, closures of schools and universities, home working and avoidance of all “non-essential” travel. On 23 March 2020, the UK government implemented strict lockdown measures in an attempt to reduce transmission of COVID-19 and halt the pandemic. People were instructed to stay at home and to avoid all “non-essential” contact with people outside of their household.

The novelty of COVID-19 placed an unprecedented demand on health and social care services [1]. Defined by the NHS as a significant emergency, healthcare workers became the heart of the unparalleled crisis of COVID-19, facing treatment challenges, responsibility for reducing the spread of infection, developing suitable short-term strategies and formulating long-term plans, all while trying to keep themselves and their families safe from exposure to the virus [2]. While huge burden was placed on frontline workers of the pandemic, healthcare leaders were tasked with the responsibility of supporting frontline workers and undoubtedly faced increased pressures.

There is universal consensus that good leadership is a requirement in healthcare [3]. Leadership is the process of influencing others to understand and agree on what needs to be done and how to do it, while facilitating both individual and collective efforts to accomplish shared objectives [4]. In clinical settings, effective leadership has consistently been identified as a core component of high quality care and healthy workplaces [5]. Leadership literature references numerous leadership models and styles, which all have their advantages and disadvantages in different contexts [6,7]. Previous research demonstrates that good leadership practice in organisations has correlations to improvements in patient care, innovation, quality and patient safety [8].

Effective leadership in healthcare is often complex, as leaders need to consider “*roles, relationships and practices that are made within contexts and through social interactions, while learning with people who share these contexts*” [9] (p. 257). These complexities are increased further by crisis situations, such as pandemics. Leaders face additional pressures and are expected to make fast decisions in line with new information from the government and other national health bodies with complex information about rapid necessary changes to care procedures such as care delivery and staff safety [10]. Several studies suggest that crisis leadership requires effective communication, a clear vision and set of values, a sense of shared social identity and trust [11,12,13]. Having confidence to trust leaders in such decision-making is a fundamental component of health care leadership; one cannot lead without trust [13]. Trust is defined as “the reliance of others and one’s self of character, ability, strength, truth, confidence, hope and credibility” [14], In confidence, there is a reliance and dependence on others in that we entrust others (i.e., leaders) to perform results that benefit the common good for all [13]. For staff, trust in leadership has been shown to establish many benefits including improved motivation, attitudes, behaviours, collaboration and co-operation [15].

Previous research suggests that healthcare leaders should concern themselves more with the realities of frontline workers to develop cultures of learning through shared values, compassion and continuous improvements [7,16]. A large body of research suggests that people tend to prefer leaders who cultivate a sense that ”we’re in this together”, giving a sense of collective self-efficacy and hope [17,18]. Several studies highlighted the importance of strong and effective leadership to support staff and drive patient-centered care throughout the pandemic [19,20,21,22,23]. Emerging research from multiple countries have revealed the importance of; effective communication, supportive management, prioritisation of health and safety, and providing adequate resource as key aspects of leadership support to healthcare workers during pandemics [24,25,26,27] A further study highlighted key themes such as the “psychological effects” and ”changes in team dynamics” of those delivering frontline emergency care services in a UK hospital. The researchers found that changes to teams dynamics affected people in different ways, resulting in psychological distress, moral injury and mental exhaustion [28]. However, much of the evidence comes from experiences of hospital staff delivering acute care, rather than those working in community settings. A study in Canada explored primary care teams’ experiences of delivering mental health care during the pandemic but did not focus on a leadership perspective [29]. Given the lack of focus on leadership styles and responses and the different set of challenges faced by frontline staff working solely in the community, there is need to explore the experiences of staff delivering care within these settings in the UK’s public health system, the National Health Service (NHS).

While leadership examples and frameworks during crises exist from military and emergency response sectors [30], there remains limited understanding of the experiences of different leadership approaches in healthcare during a pandemic. In “The Politics of Crisis Management”, Boin et al. suggest that crises are characterised by three things: ”uncertainty, urgency and threat’ [31]. In terms of COVID-19, all three of these elements were present for staff. This paper seeks to understand community-based frontline staffs” experiences of leadership in during the pandemic and the impact of leadership on their ability to work to deliver a community care service in a crisis.

## 2. Methods

A qualitative study using semi-structured interviews was conducted via telephone or video link. This paper reports results from the interviews. The Consolidated criteria for reporting qualitative research (COREQ) checklist is used for reporting methods and findings [32].

### 2.1. Setting

The study was conducted with staff from Community Mental Health Teams (CMHTs) across the North West of England, UK. Community mental health services are managed by a central locality management team. Care across the region is then divided into several ”hub” sites, each managed by a local hub manager who is responsible for the day-to-day management of healthcare staff working on the frontline.

### 2.2. Participants

Participants who worked within a CMHT at a trust in North West of England, UK were invited to take part in the study, regardless of profession. A trust is an organisational unit within the National Health Service (NHS) in England which provides medical services to either a geographical area or a specialised function (i.e., mental health services). An advertisement added to trust communications issued as the daily COVID-19 bulletin via email was distributed. Additionally, key stakeholders distributed the advertisement to CMHT staff. In this way, although staff promoted the study through the provision of the advertisement, they did not target staff personally or actively recruit them to the study.

Potential participants were asked to contact the research team directly, either by phone or email. Once the research team were contacted, participants were sent an information sheet and consent form. If they remained interested, a date was set for interview and the medium by which the interview would take place was agreed.

### 2.3. Interview Method

Interviews were conducted remotely, via video or telephone. This was viewed as the most effective way in terms of convenience and safety to collect information about sensitive topics in the workplace during the pandemic [33]. A semi-structured interview guide was developed which focused on broad experiences of CMHT staff during the pandemic such as their clinical and personal experiences, changes to working practices and implications to care. Participants were also asked about support from the trust and how the trust may provide better support in the future. The semi-structured nature of interviews meant that the leadership focus varied in each interview, with some discussing local, departmental leaders while other discussed senior leadership decision-making.

Interviews were recorded using a digital audio recorder and transcribed verbatim by independent transcribers employed by University of Liverpool. Interviews were conducted by authors AW and EP and another researcher from the wider team. All interviewers were female and were qualified to MSc level or above, with previous experience of undertaking qualitative interviews. The interviewers were matched with participants that they did not know or had not previously worked with. Participants were made aware of the interviewer’s role and research interests prior to interview commencing.

The interviews took place from 5 June–29 July 2020, approximately three months after the UK announced the first national lockdown. Interviews lasted between 24 and 100 min, with an average interview length of 47 min.

### 2.4. Data Analysis

Interview data were analysed using constant comparative thematic analysis. This approach proceeded through a series of sequential steps: close reading of the transcribed interviews followed by open coding in which the data were grouped into smaller units with codes attached [34]. After analysing the initial three interviews, an analytical coding framework was developed through consensus between the researcher analysts. Authors LB and AW independently developed codes that were derived both deductively (based on the initial coding framework) and inductively (allowing for new codes and sub-themes to emerge from the transcripts). Themes and sub-themes were discussed iteratively with the wider team until consensus agreement was reached. The developed codes were then grouped into categories within the major components of the analytical framework [34].

## 3. Results

Twenty-five potential participants expressed interest in the study. Four did not respond to the reminder email, leaving twenty-one frontline CMHT staff who took part in interview. Of the twenty-one participants, there were nine male and twelve females. Thirteen were employed as Community Mental Health Nurses (CMHNs). Other participants included social workers, physiotherapists, support workers and staff from managerial positions. Staff had worked for the trust for varying durations, ranging from one month to thirty-one years.

### 3.1. Findings

**Figure 1:**
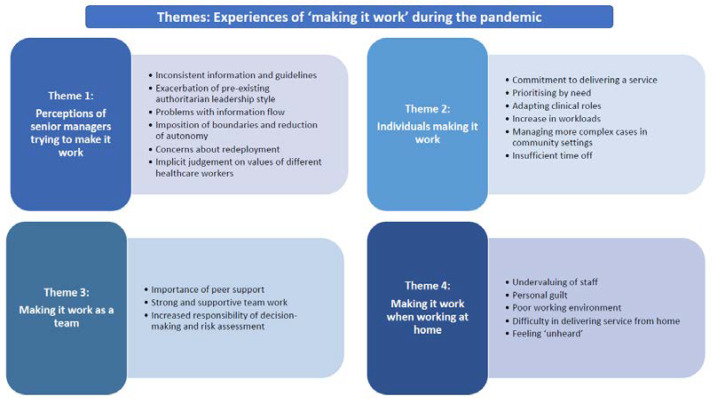
Themes: Experiences of “making it work” during the pandemic.

In their different ways, all of the staff interviewed in this study were trying to make the delivery of care to people with mental health issues “work“. “Making it work” took different forms depending on the perspective adopted as can be seen through the four themes identified; “senior trust managers trying to make it work”, “individuals making it work”, “making it work as a team”; and “making it work through working at home” (Figure 1). The title reflects the different ways in which people in the NHS organisation achieved the delivery of care during the pandemic. It was more common for staff to feel that the senior leadership asserted and mandated ways of performing their role, often through the imposition of boundaries around care delivery, while the staff on the ground adapted to their circumstances and tried to achieve service delivery within the parameters imposed by the Trust.

### 3.2. Senior Trust Managers Trying to Make it Work

Staff spoke of their perceptions of how senior Trust managers tried to make service delivery work. Staff reported that prior to the pandemic there was predominantly an authoritarian senior management system, with little flexibility and rigid structures making it difficult for frontline staff to make autonomous decisions. In the context of the pandemic, there was a perception that the same authoritarian model of decision-making was used to make care in the community happen. However, the government guidance frequently changed over the course of the data collection as more became known about COVID-19, and its impact on the NHS and wider population. Information for all NHS providers was filtered through the layers of management in different ways. In community services, there was a level of variability in the time it took for information to reach staff which meant, on occasions, what was received by staff was reported to be out of sync with National guidance.

*“what we did, we said things like, yes, it’s safe to do group work and we would tell people this at lunch time and then the Prime Minister would deliver his briefing erm an hour later saying people are no long permitted to meet in groups... so the very next morning we would have to contradict our message and so there was something about how we synchronised the advice to give people that undermined their confidence in us”*

(Respondent 015)

*“…you would go from one meeting to the next and advice was changing constantly, so you were giving staff advice one minute and it was changing the next so trying to remember all that, that was quite difficult”*

(Respondent 021)

Uncertainties about the nature of the virus, its transmission and its impact were compounded by frequent changes in the guidance for staff. Information was communicated electronically to all staff with the expectation that messages would be cascaded through the system by word of mouth. Not surprisingly, problems with information flows contributed to difficulties in service provision.

The complex relationship between senior management and frontline staff emerged as a significant theme running through this research. Staff believed that decisions were predominantly made at a senior level of the organisation and passed down to front-line staff in an authoritarian manner.

*“You’re not being asked, you’re not being informed, you’re just gonna do what we [management] say, or not do, and there’s no reasoning behind it”*.

(Respondent 013)

The content of these managerial decisions largely related to the imposition of boundaries around the work that was to be undertaken. These boundaries encompassed physical space (where people should work, who they should travel with and how), professional roles (who should do what, to whom and how) and personal and inter-personal spaces (how they should be protected through working at home/office, use of personal protective equipment (PPE). In the wake of the crisis created by the pandemic, the communication of these new boundaries was designed to minimise ambiguity and keep the service running while keeping both staff and service users safe. However, the decisions made by management were questioned by many staff who reported that they did not take account of the realities of working on the ”frontline”. These realities included issues surrounding the supply of PPE, the proportion of staff who were able to work in patient or service user facing roles, and the retraction of most support services which enabled service users and their families to remain living at home.

“*every decision that’s been made, everything’s being done without discussion, without asking anyone…, every morning, every afternoon, him and the other* *managers, they go into a side room, they’re there for a couple of hours, morning and afternoon, they come back out, decisions have been made, discussions have been had, we know nothing until something changes either in the off duty or you know, an email comes out saying this is going to happen*”

(Respondent 013)

Staff were divided about whether management directives were overly prescriptive, retracting the ability of staff to make autonomous decisions, or whether they were too opaque, requiring staff to use their discretion and get their decisions approved by local managers.

“*I don’t feel we are able to use our discretion with it to be honest with you because we’re being told that this is the trust policy, this is what we need to stick to*”

(Respondent 010)

The new ways of working imposed on staff encompassed some ideas that had been requested by staff in the past, but which had not been met with support. Such decision-making led to frustration as to why it took a pandemic to allow changes which had already been requested.

*“…now it’s been forced on them they say well why didn’t we do this in the first place!... they were just adamant that they would not let you work from home. And you know it just couldn’t be done and you know as soon as the COVID thing come, everyone’s working from home”*

(Respondent 001)

Imposing changes in working practices constituted an important aspect of the leadership response to the pandemic directed at keeping staff and service users safe. Perhaps the most difficult of changes to the working conditions of staff concerned the redeployment of staff out of the community into inpatient settings. As the extent and severity of the pandemic developed, trust senior management became concerned about staffing levels within acute inpatient wards due to staff self-isolating, shielding and general staff illness. As a result, CMHT team leaders were asked to redeploy community staff to inpatient wards.

“*It wasn’t put out there as a voluntary-as something that you could volunteer for, because we knew that no one basically would volunteer. So it came down as a management instruction from the senior management team in the local division, so that the instruction came down and then the instruction was to have a look at all your teams, look at the minimum amount of staff that you could work, that could maintain the team, and then anything that’s left over then would have to be redeployed*”

(Respondent 011)

In the interviews, staff described feeling pressure to volunteer. Staff expressed anxiety and fear about contracting the virus, transmitting the virus to their family and being redeployed as a form of retaliation for a lack of cooperation or for raising too many questions about what they were being asked to do. Not only was redeployment stressful for staff being redeployed, it created gaps in the teams working in the community. Decisions about volunteering to be redeployed to inpatient settings were made difficult because of the uncertainty early on in the pandemic about virus transmission, effectiveness and availability of PPE, and the media focus on morbidity and mortality as a result of COVID-19 infection.

*“it caused a huge amount of upset, and there was people really fearful by going back onto the wards because at that point, every night on the news, all you had was news items on the ITUs and staff wearing full PPE [personal protective equipment] and deaths and the rising death toll, and staff who were being redeployed really did feel that they were being put at more risk by being redeployed into the inpatient wards”*

(Respondent 011)

Prior to the pandemic, the place of work, hospital or community, was a matter of individual choice. In the context of the pandemic, staff were shifted across locations to meet the needs of the wider service.

The diversion of staff into inpatient settings prompted discussion about the feelings of inequality between hospital and community-based staff. There was a commonly held belief that everything from PPE to COVID-19 testing was prioritised for hospital staff while community staff received these much later in the chronology of the pandemic.

*“for us in the community, even though we’re seeing people are much as probably, you know, we’re seeing a high level of people, there still wasn’t any COVID testing for us…again, we were probably bottom of the chain you know, I think the ward staff, the mental health ward staff, they were all tested, which was rightly so. But for us in the community, we were not tested”*

(Respondent 009)

The inequalities perceived to exist between hospital and community were exacerbated on 30 July by the Secretary of State for Health, Matt Hancock, who announced in a speech at the Royal College of Physicians (Hancock 2020) that ”from now on, all consultations should be teleconsultations unless there’s a compelling clinical reason not to”. The staff in this study who continued to visit service users in their own home questioned whether there were some implicit value judgements being made about the relative value of the lives of GPs and psychiatrists versus community nursing staff.

*“what’s been really difficult as well, because doctors haven’t been going out at all to see patients only if they’ve had to, say, maybe section somebody or a very urgent review. But we’d still be expected to go out and see people... there was a little bit of kind of it was felt are they, their families more important than ours”*

(Respondent 021)

In common with the perceptions of the public, the frequent changes in government guidance which were passed onto NHS service providers were viewed as ‘incompetent’ and lacking in a clear message. Peter Sandman [35] writing about risk suggests that uncertainty is often associated by the public with incompetence and, as such, becomes open to challenge. In these interviews with staff there was a feeling that the frequency of changes to the message from the senior management Team in the Trust resulted from being **“***slapdash*” (Respondent 013) rather than from genuine uncertainties in the scientific knowledge at that time.

*“we weren’t wearing any PPE in the office at that point we were just told to social distance of course our own common sense had told us by then to do that… Then 2 weeks ago we got guidance that we were all to wear masks, if we get up from our desk and move around the office we now have to wear masks…its little too late that to me”*

(Respondent 018)

*“it’s not been their fault as such-is that the constant backtracking on decision making and that’s happened loads…, like we were hearing instructions coming from senior leadership but then at the five o’clock sort of briefings, Boris would be telling us sort of something else!* *”*

(Respondent 011)

The frontline staff in this study mostly viewed senior staff as making service provision happen at a distance through imposing boundaries on their work. However, local team managers were seen as less remote and views on how work was organised and supported varied according to the local team manager.

### 3.3. Individuals Making It Work

All of the staff reported a commitment to the care and support of service users and wanted to be able to provide them with a face-to-face service. In addition, staff wanted to support each other (explored in the next section) and often reported not wanting to leave their colleagues unsupported (Respondent 008).

*“the reason you do the job is because we want to help… that’s why we do it, to try and help and for me to say, well sorry right now I can’t do that, and it’s not so good, but yeah I’m not comfortable about it, but I don’t think I should be put in that position either”*

(Respondent 004)

*We’re trying, we’re doing the best that we can with limited resources and limited capability. There’s a lot of stuff that we’re not supposed to be doing, we get the guidelines, we do our best to follow them, of course they’re guidelines, they’re not rules so, if you need to take one or two shortcuts, for instance, so long as everything’s safe, then get on with it, you know? You’ve got to think generally, it’s the service user at the end of the day that needs the support*.

(Respondent 006)

Staff who did not need to shield were keen to continue seeing service users face-to-face but in general followed the Trust policy of only visiting people who were in absolute need.

*“…so every visit that we do as community [staff], there’s got to be a rationale for why we’re doing that, what’s the urgency to that, what’s the crisis before we can make that decision to go out and ordinarily you would have made those decisions without passing it by any management, that’s what we would have just done as community nurses in the past”*

(Respondent 010)

In some cases, the definition of what was needed by service users dictated the nature of the care to be delivered. For example, those requiring depot injections required a regular face-to-face appointment to receive their treatment. In the case of one member of staff, fetching and carrying replaced their previous role of sitting and talking to service users.

*“all I’m doing, all my role is limited to is delivering medication and prescriptions, shopping, and food bank parcels, there’s no kind of one-to-one support”.*

(Respondent 004)

Individual members of staff who continued to provide face-to-face care and support for service users often reported feeling conflicted about the nature of the service they were able to provide. There was a recognition that although the overall number of service users might not have increased, as identified above, some service users were being cared for at home with greater, more complex, needs which was intensified by the reduced number of staff available to deliver the service. The absence of capacity elsewhere in the system often resulted in staff maintaining ”risky” service users in the community although it was recognised that this was not the right location for their care.

*“There’s 3 patients, really really high-risk patients that are waiting to go in for inpatient, they have a mental health act framework in place, but because there is not a single bed available, we’re trying to manage these risky patients in the community which is increasing a lot of kind of staff pressure and stress”*

(Respondent 008)

*“The caseloads have remained the same within teams, but the workload for those practitioners who can still do face-to-face contacts, their workloads have increased, because they’ve had to take on other service users, because the other care coordinators have not been able to offer face-to-face contacts”*

(Respondent 011)

The increase in the workload of those people actually providing face-to-face care, combined with the changes in the nature of that care produced a ”task and go” approach to care.

“*so let’s say somebody was supposed to have an hour’s visit, for personal care, medication and meals, it’s then changed to task and go so basically, they went in, did whatever they had to do and get out as quick as they can. So people were having a reduction in the contact they were having each day*.”

(Respondent 002)

While some individuals appeared to be providing less contact with a service user in order to see more people, other staff reported providing a wider range of services per visit, doing shopping for instance, to compensate for the absence of other services.

*“I have had to kind of go above and beyond and I have had to adapt my depot clinic to a mobile depot clinic to go to the house in the morning as opposed to them coming in to clinic here, so it kind of has increased my case load in that respect ….so everyone’s kind of got an additional role for seeing people for the depots”*.

(Respondent 08)

Despite the increased pressure on staff there was a reluctance to discharge people when the whole landscape of support services had disappeared.

*“a lot of mine [service users] are actually at that point of discharge…at the moment I haven’t discharged them ‘cause at least they’ve got a point of contact but how long do I keep them on my caseload until I say, I’m sorry but you’re going now!”*

(Respondent 002)

In summary, staff were keen to deliver a face-to-face service to service users wherever that was possible and if they felt there was a need. Many of the staff interviewed talked about giving up their annual leave or coming off their annual leave early in order to support service users.

*“I’ve kind of very much been filling those gaps [in the service] …I haven’t had an opportunity to take any annual leave in those erm…well what is now 4 months”*

(Respondent 015)

In the next section we explore how the individual approach to making community mental health care happen operated within the wider team environment.

### 3.4. Making It Work As a Team

Some CMHT staff spoke positively about their individual experience of working during COVID-19, reporting that they felt well supported and that their needs were met through regular supervision, good communication and understanding and flexibility around changing personal circumstances as a result of the pandemic.

*“I have to say I’ve been pleasantly surprised at how well, you know, they’ve [management] adjusted to you know the new circumstances and the positivity and feeling that your work is appreciated.”*

(Respondent 001)

Although many staff talked about individual circumstances framing their decisions, there was a strong emphasis in the accounts of staff providing face-to-face care on the importance of their colleagues and the role of the team in making care happen.

*“I think we just as a team really quickly realised, you know, that we were going to have to do something before the lockdown really happened, so we did sort of address all of that, got rotas together and sort of everyone made a list of you know their vulnerable patients and things you know for the people that were on duty that would know about, so…I just felt it was quite well organised really, as a team”*

(Respondent 010)

Either as a result of good team management, strong pre-existing team structures or due to the way in which staff pulled together, there were many examples in which staff supported and ”covered” for each other.

*“I suppose what we have here, is, I suppose is a very strong team and we cover for each other, and we are very supportive of each other, and I suppose we seem to sort of manage to get through somehow which we do which I think says a lot of the people I work with most definitely, very resourceful, very resilient despite everything.”*

(Respondent 012)

*“I know we’re a good team and when I’ve needed someone to cover an injection because I’ve got other things there is always someone on my team, we’re a really good team so we can kind of support each other in that respect...”*

(Respondent 008)

Peer support during the pandemic was seen by staff as very important at many levels. While covering for each other was a feature of managing the workload, it was in the absence of peer support that the value of colleagues became apparent. A lack of regular peer support produced anxiety through the inability of staff to ”sense check” their decisions. Being isolated and alone in relation to the management of people with mental health issues in the community increased feelings of responsibility of staff with regard to decision-making and risk assessment.

*“you actually miss that contact don’t you, with your team members…I mean I always find that a lot of my supervision is really informal, you know, you come back after a visit and you chat with one of your colleagues and say, have I done that right, have I made the right decision and you haven’t got that so it does feel quite isolating I think”*

(Respondent 010)

### 3.5. Making It Happen through Working at Home

While there were staff who were working face-to-face with service users who reported feeling isolated, the experience of working from home generated the strongest accounts of isolation. Some staff reported feeling undervalued, unappreciated and “*forgotten about”* (Respondent 001).

*“people who are in the shielding group, it was my understanding that regular contact should be made from somebody. I’ve, the only thing which has been keeping me up to speed with what’s happening with [Trust] is blogs and the Facebook pages (laughs)”*

(Respondent 014)

Staff who were shielding due to pre-existing health conditions also reported struggling with their own concerns and their own guilt that they were not working on the frontline.

*“People that haven’t been able to fulfil their role through no fault of their own through health issues or whatever you know have probably struggled more”*

(Respondent 018)

Staff were also concerned that their invisibility might be taken for not working.

*“I worry about people thinking that I’m not working my full hours or might be having a fine old time at home sort of thing…but I think that’s just my own concerns, not like management have been very strict over that”*.

(Respondent 010)

The experience of working from home varied enormously. The presence of children, poor technology and internet connections and the use of telephone contact for mental health assessments, all made working from home a stressful experience for many.

*“So I think that’s been more of a problem so I think people are holding back really on sensitive information that normally they would have shared with us”*

(Respondent 010)

*“I just didn’t see the, I didn’t see what good it was going to do, not seeing people and just phoning them, but as the weeks have gone on, I mean I’m phoning people I’ve never even met which is really hard ‘cause at least if you’ve met them, you can sort of see their home environment and you know what their care is like, but some of these people I’ve never actually met so you just phone them knowing nothing”*.

(Respondent 002)

The physical environments in which people were working also created additional difficulties.

“*I know it’s my own fault, but I sit on the settee, which obviously isn’t a good place to sit all day long doing your work, but if you sit on the kitchen table that’s also quite uncomfortable*”

(Respondent 002)

“*I just find it so boring just sat here on my own.*”

(Respondent 002)

However, a few staff reported very positive experiences of being supported by team members despite working from home.

“*I’ve got really supportive* *managers* *and really supportive other colleagues, we have regular check ins with me, how are you doing? are you sure you’re ok? My manager and line manager both message me regularly, we’ve got a WhatsApp group that we chat on, we have emails off each other*.”

(Respondent 003)

Staff also reported that the positive response from service users to receiving a phone call enabled them to feel that the service they were delivering was worthwhile.

“*But I think I have got used to it and I have noticed that they do appreciate it and they need it, even if it is just a phone call*.”

(Respondent 002)

In the context of restricted face-to-face contacts most of the work was transferred online and meetings took place via video conferencing. While the opportunity to meet virtually was seen as better than not meeting at all, it was not always easy for people to get their voices heard and the nature of the meeting often prevented people raising questions that might have been asked in a face-to-face context.

*“people are feeling the Zooms aren’t appropriate to raise questions that they want to ask”*

(Respondent 013)

## 4. Discussion

This study of twenty-one frontline community mental health workers in the North West of England confirms that despite the difficult circumstances arising from the COVID-19 pandemic, staff tried to deliver a safe and effective service to people with mental health needs. However, a strong thread running through the accounts of staff related to the way in which frontline staff felt excluded from the critical decisions about how care should be delivered. They experienced the imposition of boundaries from the Trust senior leadership with limited scope to interpret the Government guidance on matters which affected their delivery of frontline services.

In this study staff reported that an already authoritarian approach to managing staff became much more explicit during the COVID-19 crisis. While there might have been good reasons for this, these reasons were not explicitly communicated to staff. While healthcare providers often operate in a state of tension between demands for care and available resources [36], a healthcare crisis may exacerbate this tension, generating imbalances which lead decision-makers and frontline staff to adapt their activities, improvise solutions and make compromises. These adaptations lead to divergences between the “work as done” (i.e., by frontline staff) and the “work as imagined” (i.e., procedures and protocols outlined by Trust leaders) [37]. Senior leaders tried to make services work within the protective scaffolding provided by the Government legislation and guidance. However, the realities faced by frontline staff did not always match with the directions from the Trust’s leadership team. There were, for example, problems in working from home, delays in receipt and use of PPE and staffing issues, which hindered the ability of staff to provide a service as they would have liked. Trying to follow the Trust’s guidance to protect themselves while fulfilling their professional obligations caring for vulnerable service users was stressful for staff. Anderson [38] suggests that organisations should focus on enhancing their adaptive capacity during crisis situations, where situations and circumstances cannot always be anticipated. Therefore, flexibility beyond rigid protocols and procedures may be required to make service delivery work [38].

Traditionally, and particularly in emergency situations, healthcare leadership practices have been associated with the military paradigm of “command and control” [7]. While there are many ways to conceptualise leadership styles, Todd Dorman suggests that historically “a hierarchical approach to management was not only beneficial but required” in those circumstances where there was a need to respond to emergencies [39] (p.196). Dorman [39] identified some of the drawbacks of this approach in the context of ICU work including; it does not get the best out of our team; it fails to keep the needs of patients and their family members central to decision-making, and; when something goes wrong there is a need to assign blame [39]. Interestingly, Dorman also identifies the way in which authoritarian styles of leadership fail to engage staff [39]. By contrast more modern forms of leadership are often described as inverting the more traditional power structures such that frontline healthcare staff are trained and expected to take informed evidence-based decisions at an operational level [40].

Holge-Hazleton et al. [7] suggest that in reality, a “command and control” approach to leadership has a negative impact on frontline leaders who have the responsibility for implementing new practices, procedures and behaviours [7]. This was highlighted in the difficulties experienced in transferring staff to work in inpatient settings. The study identified perceptions of disparities between community and hospital services with hospital-based staff being given priority access to PPE and testing. Further, the importance of maintaining staffing levels in hospital, despite the attrition of staff working in the community seemed to emphasise to staff the importance of the hospital over the community. Given that the predominant concern recorded across all healthcare staff groups in all pandemics was becoming infected with the virus themselves or infecting family members [41], it is perhaps not surprising that community staff found the prospect of being re-deployed extremely stressful [42]. In prior pandemic situations, such as the SARS experience in Toronto in 2003, there were similar feelings among staff about the relationship between duty and risk, with questions being raised about the balance between self-regard and the pursuit of professional duties, despite the threat to self [43]. Benedetti writing with reference to COVID-19 suggests that health care providers have some degree of obligation to care for patients with transmissible diseases based on principles such as their professional codes of conduct/oaths [44]. However, Benedetti highlights the need for healthcare leaders to employ risk-reducing strategies to allow healthcare workers a safe working environment, allowing them to continue to deliver a service while supporting their own protection [44].

The study demonstrates the importance of effective crisis communication in maintaining staff confidence and equipping them with necessary knowledge and attitude to make it work when delivering safe and effective care in a crisis. Emergency risk communication is defined by the WHO [45] as an intervention ”to enable everyone at risk to take informed decisions to protect themselves, their families and communities against threats to their survival, health and well-being”. As a result, risk communication has been the subject of evidence-based guidance. Studies in the field of emergency risk communication suggest that the timing and method of communicating messages during a crisis are critical to whether recommended behaviours are followed or not [46,47,48]. In this study, it was unclear whether there had been much consideration given to the way in which messages were communicated to staff. Staff perceived an ever-changing flow of messages from the Trust. In part, this reflected the way in which Government guidance changed, but it was received by staff as “slapdash”, with an oversaturation of competing messages. It is likely that the desire to communicate key messages outweighed consideration of the ways in which messages could be targeted at different groups of staff. A study of problematic pandemic-related situations that healthcare teams in a University hospital in Switzerland faced suggested institutions “could support their managers by providing the resources to help them improve their communication skills, transparency, empathy and team management”. In doing so, Juvet et al. [37] assert employees’ feelings of misinformation, uncertainty, inequity, or exhaustion can be countered and their resilience improved [37]. These findings resonate with research conducted during previous health crises such as SARS [49] and Ebola [30,50] where the type and amount of communication delivered to clinicians made it difficult to assimilate.

Our study sheds light on the difficult pressures that staff are dealing with as part of the pandemic response and the support received from healthcare leaders. While this study captures the leadership experiences of a range of community mental health staff working during the first wave of the pandemic, it does have several limitations. The study was based in NHS community care but did not include all point-of-care staff including occupational therapists, healthcare assistants or GPs and psychiatrists. Additionally, our sample is only representative of one community mental health workforce in the North West of England. Due to the sample size it is not possible to share information about the clinical role of each participant without risking de-anonymisation of the data. Finally, the study was conducted during the first wave of the pandemic only and therefore does not explore subsequent peaks or the longer-term impacts of the pandemic on leadership experiences. Future research should address the issue of how leadership practices can be established which are flexible enough to adapt to crises while allowing frontline staff to feel empowered to deliver the optimal service possible.

## 5. Conclusions

Recovery from the pandemic is a potential opportunity to establish new ways of working, including leadership. While in this study, there was evidence of a “command and control” leadership style being implemented in response to the crisis, we question whether this approach is appropriate. Adopting such an approach without the necessary infrastructure to support it, resulted in tension within the team due to difficulties in “making it work”. The study raises the question of whether it would be beneficial for those working on the frontline to be more involved in decisions about how policy is translated into practice, particularly during a crisis. Effective crisis communication is essential to help maintain staff confidence and equip them with necessary knowledge and attitude to make it work when delivering safe and effective care in a crisis. Applying lessons from this study at various levels of management will help to support frontline staff, both psychologically and in strengthening care delivery.

## Data Availability

The data presented in this study are available on request from the corresponding author. The data are not publicly available due to privacy reasons.
